# Inducing Non-genetically Modified Induced Embryonic Sertoli Cells Derived From Embryonic Stem Cells With Recombinant Protein Factors

**DOI:** 10.3389/fcell.2020.533543

**Published:** 2021-01-25

**Authors:** Chenze Xu, Ali Mohsin, Yanxia Luo, Lili Xie, Yan Peng, Qizheng Wang, Waqas Ahmed, Haifeng Hang, Yingping Zhuang, Meijin Guo

**Affiliations:** ^1^State Key Laboratory of Bioreactor Engineering, East China University of Science and Technology, Shanghai, China; ^2^Engineering Research Centre of Processes System, Ministry of Education, East China University of Science and Technology, Shanghai, China; ^3^Institute for Stem Cell and Regeneration, Chinese Academy of Sciences, Beijing, China

**Keywords:** embryonic stem cells, Sertoli cells, non-genetically modified, recombinant endogenous protein factors, lentiviral transduction

## Abstract

Embryonic Sertoli cells (eSCs) possess multiple supporting functions and research value in gonadal development and sex determination. However, the limitation of acquiring quality eSCs had hindered the further application. Herein, we successfully derived non-genetically modified (non-GM)-induced embryonic Sertoli-like cells (eSLCs) from mouse embryonic stem cells (ESCs) with a TM4 cell-derived conditioned medium containing recombinant endogenous protein factors *Sry*, *Sox9*, *Sf1*, *Wt1*, *Gata4*, and *Dmrt1*. These eSLCs were determined through morphology; transcriptional expression levels of stage-specific, epithelial, and mesenchymal marker genes; flow cytometry, immunofluorescence; and immunocytochemistry and functionally determined by coculture with spermatogonia stem cells. Results indicated that these eSLCs performed similarly to eSCs in specific biomarkers and expression of marker genes and supported the maturation of spermatogonia. The study induced eSLCs from mouse ESCs by defined protein factors. However, the inducing efficiency of the non-GM method was still lower than that of the lentiviral transduction method. Thus, this work established a foundation for future production of non-GM eSLCs for clinical applications and fundamental theory research.

## Introduction

Sertoli cells, as the main supporting cells of testes, have important roles in male determination, male gonadal development, and normal testicular functioning (Skinner and Griswold, 2005; Carre and Greenfield, 2016; Gunes et al., 2016). They possess high clinical importance due to their immunosuppression function for transplantation application (Luca et al., 2018). Sertoli cells have been widely applied in fundamental research such as facilitating the survival and proliferation of neurons (Deng et al., 2014), endothelial cells (Fan et al., 2011), and hepatocytes (Zeller et al., 2017); differentiation and growth of mesenchymal stem cells (Monfared et al., 2016); and secretion capability of islets (Li et al., 2011). Moreover, it is generally hard to achieve sperm maturation of spermatogonial stem cells (SSCs) *in vitro* without Sertoli cells as feeder cells (Geens et al., 2011; Baazm et al., 2017; Griswold, 2018).

Sertoli cells can be divided into three stages: embryonic Sertoli cells (eSCs), immature Sertoli cells, and mature Sertoli cells (Wilhelm et al., 2005; Nel-Themaat et al., 2011; Chojnacka et al., 2016). Mature Sertoli cells have been widely studied as feeder cells since they are most easily extracted from donor mouse testes among the three stages (Franke et al., 2004; Skinner and Griswold, 2005; Yue et al., 2006; Fan et al., 2011; Geens et al., 2011; Li et al., 2011, 2012; Monfared et al., 2016). However, mature Sertoli cells are mitosis inactivated and hardly cultured for more than 2 weeks. To address this problem, Sertoli cells were genetically modified (GM) to produce TM4 cell lines as semipermanent Sertoli-like cells (Tanphaichitr and Fitzgerald, 1989). Still, they are not the perfect solution. In coculture systems, TM4 cells can rapidly overgrow on account of their characteristic of being nearly infinitely mitotic-activated. Then, eSCs could be an alternative. In male gonad, eSCs secrete multiple functional factors including follicle-stimulating hormone (FSH), anti-Mullerian hormone (AMH), and insulin-like growth factor 1 (IGF1) (Barrionuevo et al., 2011; Chojnacka et al., 2016). Compared with mature Sertoli cells and TM4 cells, some evidences indicated that eSCs could be superior in facilitating the maturation and proliferation of SSCs (Nel-Themaat et al., 2011; Baazm et al., 2017; Griswold, 2018). Therefore, eSCs had shown high potential as feeder layers. In other ways, studies on eSCs would hold promise to reveal the mechanism of sex determination and male gonadal development since they are the first male-specific cells generated during mammalian embryogenesis (Barrionuevo et al., 2011; Nel-Themaat et al., 2011). Thus, eSCs are of great value for fundamental theory research and future clinical application.

However, eSCs ethically cannot be obtained from the human embryo. Moreover, the cell population of eSCs is very limited in each mouse male embryo and hard to meet the demand of experimental studies (Barrionuevo et al., 2011; Baazm et al., 2017). Thus, some research was aimed to provide the solution. Recently, a new approach produced intermediate mesoderm (IM)-derived cells from mouse embryonic stem cells (ESCs) by using some supplements including retinoic acid and Activin A, and then induced the IM-derived cells into embryonic Sertoli-like cells (eSLCs) via some recombinant protein factors including follicle-stimulating hormone (FSH) (Bucay et al., 2009; Oeda et al., 2013; Seol et al., 2018). However, the differentiation fate and molecular mechanisms in this approach were still unclear. Moreover, another research successfully created eSLCs reprogrammed from fibroblasts by overexpression of 5 factors, which indicated that these development-related factors, *Wt1*, *Gata4*, *Sf1*, *Sox9*, and *Dmrt1*, could be significant to the generation of eSCs (Buganim et al., 2012). Thus, based on these approaches of inducing eSLCs, and the established theory of differentiation fate and molecular mechanism of derivation of eSCs, our former work successfully derived induced eSCs from mouse ESCs by lentiviral transduction for overexpression of *Sry*, *Sox9*, *Sf1*, *Wt1*, *Gata4*, and *Dmrt1* (Xu et al., 2019). The population of eSCs reached 24% in the whole cell population (1 × 10^4^ cells/cm^2^) and over 55% with pebble-like colonies (PCs) removed in 5 weeks post transduction. We proposed that these 6 factors respectively play important roles in different developmental stages such as the generation of somatic cells of coelomic epithelium, *Sf1*-positive precursor cells, and eSCs (Koopman et al., 1990; Hacker et al., 1995; Kent et al., 1996; Morais da Silva et al., 1996; Karl and Capel, 1998; Raymond et al., 2000; Hammes et al., 2001; Tevosian et al., 2002; Chaboissier et al., 2004; Qin and Bishop, 2005; Bouma et al., 2007; Sekido and Lovell-Badge, 2008). However, these induced eSCs were not perfect experimental subjects since they had been modified genetically. Thus, we intended to establish a novel non-GM method of efficiently inducing eSLCs from ESCs.

In this work, the 6 factors *Sry*, *Sox9*, *Sf1*, *Wt1*, *Gata4*, and *Dmrt1* were respectively synthesized as proteins in HEK293 cells and applied to produce an inducing conditioned medium. After a 30-day induction, non-GM eSLCs were generated. However, the cell yield of induced eSLCs was still low. Thus, we prepared the ESCs via producing a TM4-conditioned medium to improve their survival and proliferation. Then, non-GM eSLCs were induced more efficiently. These induced eSLCs occupied 10% of the whole cell population (1 × 10^4^ cells/cm^2^), with 30% of PCs removed. By analyzing transcriptional expression levels through quantitative real-time PCR (qPCR), observing cellular morphology changes under the microscope, and determining specific markers through immunofluorescence (IF), immunocytochemistry (ICC), and flow cytometry (FCM), we determined the generation process from ESCs to eSLCs by protein factors and lentiviral transduction. In comparison, the eSLCs’ inducing efficiency by lentiviral transduction (24%) was higher than by protein factors (10%). Thus, we proposed the potential barriers in generating non-GM eSLCs. Conclusively, these non-GM eSLCs could provide experimental material for future theory research and clinical application.

## Materials and Methods

### Preparation of Lentivirus, Plasmids, and Protein Factors

Sequences of *Sox9* and *Dmrt1* were purchased from Tet-on lentiviral plasmids from Addgene (United States) (Supplementary Table S1). Sequences of *Wt1*, *Gata4*, and *Sf1* were cloned from cDNA reverse-transcription products of mRNA from mouse embryos and testicular extract and then selectively amplified by PCR. The *Sry* gene was totally synthesized. Primer design is listed in the Supplementary Table S2. Then, the 6 target genes were respectively integrated into each pcDNA 3.1+ vectors between the restriction enzyme sites *BamHI* and *EcoRI* and constructed into 6 plasmids (*Sry*-pcDNA; *Sox9*-pcDNA; *Sf1*-pcDNA; *Wt1*-pcDNA; *Gata4*-pcDNA; *Dmrt1*-pcDNA). Then, these plasmids were amplified in *DH5*α competent cells, recovered by Endo-Free Mini Plasmid Kit II (TIANGEN, China), and separately transfected into three type of cell lines (HEK293, CHO, and TM4 cells) by Lipofectamine 3000 (Thermo, United States).

**FIGURE 1:**
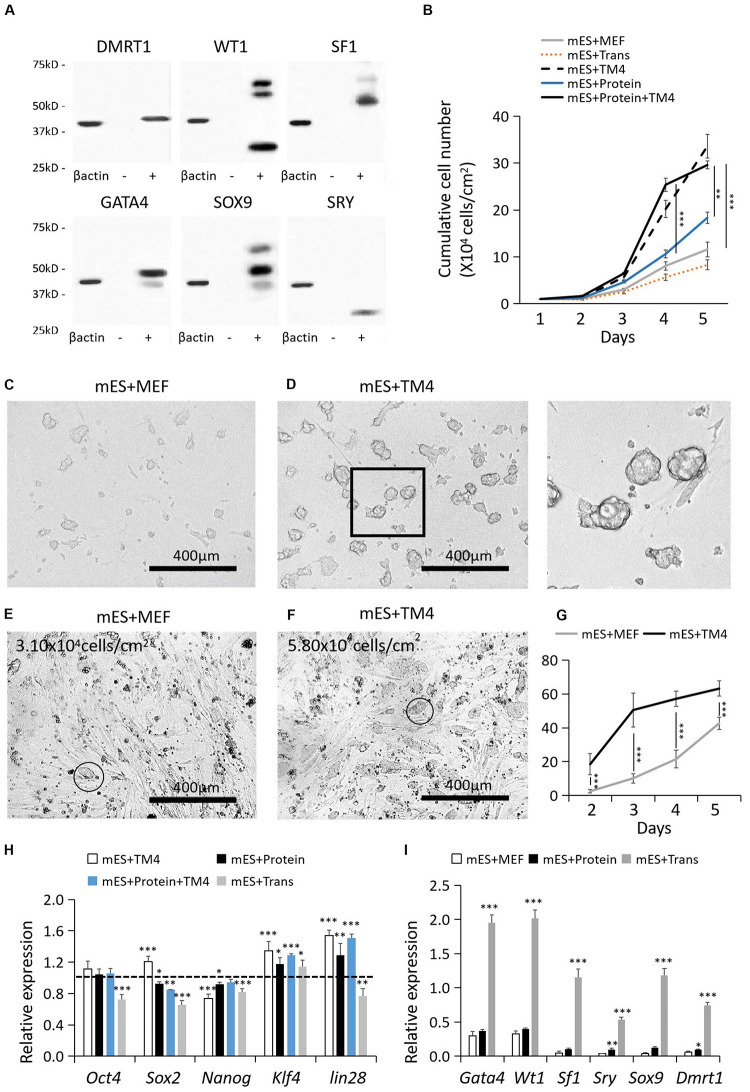
Cell recovery treated with proteins, lentiviral infection, and TM4 cells after cryopreservation. **(A)** Recombinant protein factors produced by HEK293 cells were detected by WB. The loading quantity of samples was adjusted according to β-actin. “-” represented HEK293 cells of the control group. “+” represented HEK293 cells transduced with target genes. **(B)** Cell populations of different test groups were counted in the first 5-day. Cells in mES+MEF (control group) were cultured with MEF feeder layers. In group mES+Trans, ESCs were transduced with the six factors by lentiviral infection. In group mES+TM4, ESCs were cultured in TM4-conditioned medium. In group mES+Protein, ESCs were cultured in medium containing the six protein factors. In group mES+TM4+Protein, ESCs were cultured in TM4-conditioned medium containing the six protein factors. Results were expressed as mean ± SD (*n* = 3 independent experiments). The PCs from **(C)** group mES+MEF and **(D)** group mES+Protein+TM4 were blown off by a pipette and observed under a light microscope. Scale bar = 400 μm. In panel **(D)**, the enlarged image is shown on the right. At day 3, optical images indicated the cells in panel **(E)** group mES+MEF and **(F)** group mES+Protein+TM4. Some PCs were marked with a circle. **(G)** The number of PCs viewed under a microscope per sight were counted. The amplification of the microscope was 100×. Results were expressed as mean ± SD (*n* = 3 independent experiments). **(H)** qPCR results show changes in pluripotency markers, *Oct4*, *Sox2*, *Nanog*, *Klf4*, and *lin28*, after 5 days of culture among different groups in gene expression relative to expression of the control group (mES+MEF). Results were expressed as mean ± SD (*n* = 3 independent experiments). The dotted horizontal line represents the gene expression level of each pluripotency marker in mES + MEF. Asterisks indicate statistical significance of differences in the mean gene expression according to the control group (mES+MEF) (^∗^*P*-value <0.05, ^∗∗^*P*-value < 0.01, ^∗∗∗^*P*-value < 0.001). **(I)** qPCR results show changes in 6 test factors after 5 days of culture after 5 days of culture among groups mES+MEF, mES+Protein, and mES+Trans in gene expression relative to expression of β-*actin* in self-comparison. Results were expressed as mean ± SD (*n* = 3 independent experiments). Asterisks indicate statistical significance of differences in the mean gene expression relative to the control group (mES+MEF).

After 48–72 post transfection, the protein-expressing cells were collected for WB. The recombinant proteins were labeled by corresponding antibodies listed in Supplementary Table S1. The recombinant protein factors were isolated by protein SDS-PAGE electrophoresis, recovered by Micro Protein PAGE Recovery Kit (Sangon Biotech, China), and renaturated by ProMatrix (Thermo, United States). Protein quantification was performed by Pierce^TM^ BCA Protein Assay Kit (Thermo, United States). According to the results of WB, HEK293 cells had the best protein expression capability which was applied to produce these protein factors (Figure 1A). Some of the non-singular bands of “+” results could be resulted from the changes in protein secondary structure or partial protein degradation. WT1 presented a nonspecific WB result, which also could be a result of its multiple isoforms.

To acquire lentivirus loading of the gene sequence of *Sox9*, *Sf1*, *Wt1*, *Gata4*, and *Dmrt1*, the plasmids were purchased from Addgene (United States) (FUW-TetO-*Sox9*, FUW-TetO-*Wt1*, FUW-TetO-*Gata4*, FUW-TetO-*Sf1*, and FUW-TetO-*Dmrt1*). *Sry* gene sequence was integrated into the FUW-TetO vector between the restriction enzyme sites *BamHI* and *EcoRI*, and constructed into the FUW-TetO-*Sry* plasmid. These constructed plasmids were amplified in *DH5*α-competent cells and recovered by EndoFree Mini Plasmid Kit II (TIANGEN, China).

HEK293T cells were cultured in Opti-MEM (Gibco, United States). Following the manufacturer‘s instructions, each group of HEK293T cells was separately transfected with one of the 6 lentiviral plasmids of 1.0 μg (FUW-TetO-*Sox9*, FUW-TetO-*Wt1*, FUW-TetO-*Gata4*, FUW-TetO-*Sry*, FUW-TetO-*Sf1*, or FUW-TetO-*Dmrt1*) and respectively co-transfected with 0.75 μg psPAX2 plasmid and 0.25 μg pMD2.G plasmid (Addgene, United States) by Lipofectamine 3000 (Thermo, United States) (Supplementary Table S4). The supernatant was collected after 48–72 h of post-transfection and concentrated with Lenti-Pac^TM^ Lentivirus Concentration Solution (GeneCopoeia, United States), followed by its storage at –80°C for later use.

### mES Cells and Culture

The mouse ESCs used in the current study were derived from the R1/E cell line (male gender, 129X1 × 129S1). Mouse embryo fibroblasts (MEFs) were derived from Kunming white mice between 12.5 and 13.5 *dpc* (days post coitum). Both the cells were obtained from the Chinese Academy of Sciences cell bank (Shanghai, China).

Each culture flasks were coated with Matrigel Matrix (Corning, United States). MEFs were treated with mitomycin C to inhibit mitosis and then seeded on flasks as feeder layers. After 12–24 h, ESCs were recovered from nitrogen cryopreservation using an ESC complete culture medium composed of DMEM with 12.5% fetal calf serum (FBS), 0.11 g/L sodium pyruvate, 0.30 g/L L-glutamine, 1.5 g/L sodium bicarbonate, 0.5 g/L HEPES, 50.0 μmol β-mercaptoethanol, 1× nonessential amino acids (NEAA), and 10^3^ U/mL leukemia inhibitory factor (LIF). The culture medium was replaced every day.

To produce a TM4 conditional medium, TM4 cells were cultured in DMEM medium with 5% FBS until their confluence reached 70–80%. Then, these cells were washed with PBS for 3 times. The medium was replaced with only DMEM medium. In 36 h, the culture medium was collected and concentrated by ultrafiltration devices consisting of Ultracel-3 (3 kDa) with Amicon Ultra-15 (Millipore, United States). For culturing ESCs, the TM4 conditioned medium was composed of the concentrated solution of 500 ml TM4 cells’ culture medium and 500 ml ESC complete culture medium. Then, this mixed medium was supplemented with FBS, β-mercaptoethanol, NEAA, and LIF to maintain the same concentration as in the ESC complete medium. The TM4 conditioned medium was applied in groups mES+TM4 and mES+Protein+TM4. For the ESC differentiation experiment, the concentrated solution of 500 ml TM4 culture medium was mixed with ESC complete culture medium without LIF and β-mercaptoethanol. This TM4 conditioned medium was applied in groups mES+P+TM4 and mES+T+TM4.

In comparison of the different culture conditions after ESCs were recovered from cryopreservation, ESCs were divided into 5 groups, mES+MEF, mES+Trans, mES+TM4, mES+Protein, and mES+Protein+TM4 (3 parallel samples per group). mES+MEF was ESCs with an MEF feeder in mES complete culture medium. mES+Trans was mES+MEF in mES complete culture medium transduced with 6 inducing factors by lentiviral infection. mES+TM4 was ESCs in TM4 conditioned medium. mES+Protein was mES+MEF in the medium containing 20 ng/ml of 6 inducing factors. mES+Protein+TM4 was mES+Protein in TM4-conditioned medium.

### Differentiation Strategy

In differentiation experiments, test groups were divided into 9 groups, mES+MEF (Control group), mES+Protein, mES+P+TM4, mES+P+HEK293, mES+P+CHO, mES+Trans, mES+T+TM4, mES+T+HEK293, and mES+T+CHO (3 parallel samples per group). Group mES+Protein was ESC cultured in the differentiation medium containing 6 protein factors of 20 ng/ml concentration each. Groups mES+P+TM4, mES+P+HEK293, and mES+P+CHO were mES+Protein respectively cultured in TM4, HEK293, or CHO conditioned medium. HEK293-conditioned medium and CHO-conditioned medium were produced with the same procedures of TM4-conditioned medium. Results indicated that TM4-conditioned medium had the best performance and applied for culture medium for inducing eSLCs. Culture media were replaced every 2-day. Cell passages were performed when cell confluence reached over 80%. Cell dissociation was conducted using collagenase I (Gibco, United States) for the first 10-day and trypsin-EDTA after day 10. In each test group, 5 parallel samples were performed and the results of maximum and minimum values were removed.

### qPCR Analysis

Total RNA from test groups was isolated using Invitrogen^TM^ TRIzol^TM^ (Thermo, United States), then had reverse transcription by a PrimeScript^TM^ RT Reagent Kit with gDNA Eraser (Perfect Real Time) (TAKARA, Japan). qPCR was performed with SYBR Premix Ex Taq^TM^ II (Tli RNase H Plus) (TAKARA, Japan) according to the manufacturer’s instructions on a CFX96 Touch qPCR System (Bio-Rad, United States). Primer design is listed in Supplementary Table S3.

### IF and ICC Analysis

The cell samples being fixed with 4.0% paraformaldehyde solution (10–30 min) were perforated on a membrane by Triton-X 100 (0.1%, for less than 10 min) and washed with PBS for three times (10 min per wash). Later, they were blocked with 5% bovine serum albumin (BSA) for 30 min and incubated with antibodies and DAPI (Sigma, United States) according to the manufacturer’s instruction. Followed by being washed with PBS as above, the samples were incubated with secondary antibodies for 20–30 min and then prepared for observation under an EVOS FL Auto Imaging System (Life Technologies, United States). The antibodies used in this work are listed in Supplementary Table S5.

### FCM Analysis

Cell samples were dissociated by 0.25% trypsin-EDTA and washed with PBS. If it is for detecting intramembrane markers, cell samples were treated with 4% paraformaldehyde solution for fixation (10–30 min) and then perforated on membrane by Triton X-100 (0.1%, for about 10 min), later washed again with PBS. The cell samples were resuspended in a 100-μL volume of DMEM medium in a concentration of 1 × 10^6^–10^7^ cells/mL. Matched controls of antibodies for FCM were applied according to the manufacturer’s instructions using a FACSArial system (BD Biosciences, United States). The Quad was set according to control group (mES+MEF) and isotype antibodies. Main antibodies are listed in Supplementary Table S6.

### SSC Isolation

Six comatose 2-week-old male Kunming white mice were prepared for SSC isolation. First, these mice were injected with 1% pelltobarbitalum patricum (10 mg/mL) for anesthetization according to each individual mouse (45 mg dose/kg weight). After being confirmed unconscious, these mice were executed by breaking the neck. Their abdominal cavities were surgically opened to expose the testes and then removed with tweezers.

These testes were dissociated in 0.1% collagenase type I (Gibco, United States) for 15 min under 37°C and then transferred into a new plate with 0.1% collagenase type I (Gibco, United States). The used digestive solution was collected for further use. These steps were repeated twice.

The dispersive cells in the digestive solution were centrifuged and then separated by Percoll inconsecutive density gradient centrifugation (Koh et al., 2004). Percoll gradients (Solarbio, China) were made into 8 concentrations: 65, 50, 40, 36, 33, 30, 25, and 20% in DMEM medium. After centrifuged (800 *g* for 30 min), fractions of 33 and 36% Percoll density were collected. The solution was centrifuged (800 *g* for 10 min) and washed with DMEM medium. These steps were repeated for twice. SSCs were acquired as the centrifuged product.

In mouse experiments, six male mice had operation. All experimental operations were in accordance with laws, regulations, and local ethical requirement in China.

### SSCs Differentiation and Coculture With Non-GM eSLCs

The SSC differentiation medium was composed of DMEM medium with 10% FBS containing 1 × 10^–6^ mol/L RA, 5 × 10^–3^ mol/L L-ascorbic acid, 1 × 10^–4^ mol/L testosterone (TTE), 1 × 10^–4^ mol/L dihydrotestosterone (DHT), 10 ng/mL nonessential amino acid (NEAA), and 5 mg/mL insulin.

Induced non-GM eSLCs were collected by FasL antibody (eBioscience, United States) from group mES+P+TM4 at 30-day by an FCM sorter. To coculture with SSCs, these eSLCs were treated with mitomycin C and planted on T-flasks as feeder layers. Then, SSCs were seeded on the eSLC feeder layers in an SSC differentiation medium (3 parallel samples). In the control group, SSCs were separately seeded on T-flasks in the SSC differentiation medium (3 parallel samples). After a 1-week culture, the samples were detected by IF and ICC.

### Statistical Analysis

In experiments replacing MEF with TM4 cells, test samples were cultured in T25 flasks with 5 parallel samples. A maximum value and a minimum value were removed in each test group. In qPCR, the result was the average of 3 tests for each sample. In the differentiation experiment, each test group was cultured in T25 flasks with 5 parallel samples. A maximum value and a minimum value were removed in each test group. The complete procedures were successively repeated three times. Error bars represent ± SD. Reliable data meet the condition σ (standard deviation)/μ (mean value) < 10%. Experimental data are reported as mean ± SD.

Statistical significance was evaluated by one-way ANOVA by Scheffe post hoc tests with SPSS software. *P*-values <0.05 were considered statistically significant; a *P*-value <0.01 had great significant statistical difference; and a *P*-value <0.001 had extreme great significant statistical difference.

## Results

### TM4 Conditioned Medium Improved ESCs’ Aggregation and Proliferation and Potentially Maintained Their Pluripotency

Generally, mouse ESCs were cultured on MEF feeder layers. However, ESCs still had poor cell resuscitation rate, growth rate, and easily lost pluripotency over time (Wiles, 1993; Desbaillets et al., 2000). Furthermore, the quality of MEFs feeder layers was unstable in different donor sources. These limitations hamper their application of large-scale production of ESCs. Here, we proposed to add the concentrated supernatant of TM4 cells for improving the ESC culture. TM4 cells, a genetically engineered cell line of Sertoli cells, are capable of being cultured for dozens of passages and secrete trophic proteins to facilitate other cells including nutriment retinol binding protein (URBP), tissue plasminogen activator (PLAT), transferrin (TRF), FSH receptors, androgen receptor (AR), and progesterone receptor (PR). As such, the concentrated supernatant of TM4 cells potentially has positive influence on the growth of ESCs.

In 5-day culture, the cell growth of group mES+TM4 (ESCs cultured on MEFs in TM4 conditioned medium) increased by nearly 3 times higher than that in the control group (mES+MEF, i.e., ESCs cultured on MEFs) (Figure 1B). Thus, the TM4-conditioned medium could have improved the proliferation of ESCs after cell recovery from liquid nitrogen. The cultured ESCs generally form into PCs. On the first day, the formed PCs had not solidly attached to the culture surface. Thus, the PCs of groups mES+MEF and mES+TM4 were blown off by a pipettor and transferred to other plates for observation. Under a microscope, the ESCs of mES+TM4 formed more and bigger PCs than ESCs of mES+MEF (Figures 1C,D). At day 3, more PCs were observed in group mES+TM4 than in group mES+MEF (Figures 1E,F). In the first 5-day, the number of PCs observed under a microscope per sight (100× amplification of microscope) was larger in group mES+TM4 (Figure 1G). Thus, the TM4-conditioned medium could have promoted the cell aggregation of ESCs. Generally, ESCs can hardly survive or proliferate as dispersed cells. Therefore, it is speculated that the ESCs grew much faster in mES+TM4 than in mES+MEF mainly because the TM4-conditioned medium had improved their aggregation. To evaluate the cell state of the ESCs, some pluripotent marker genes, *Oct4*, *Sox2*, *Nanog*, *Klf4*, and *lin28*, were transcriptionally determined by qPCR (Figure 1H) (De Los Angeles et al., 2015). The results were expressed relative to the gene expression of mES+MEF (control group). The expression levels of *Oct4*, *Sox2*, *Klf4*, and *lin28* were increased in mES+TM4 relative to that in mES+MEF. *Oct4*, *Sox2*, *Nanog*, and *lin28* were expressed in lower level in mES+Trans. *Klf4* was expressed higher in all the test groups relative to mES+MEF. These results implied that the treatment of ESCs with the TM4-conditioned medium or recombinant factors had no obvious negative influence on the pluripotency of ESCs. However, lentiviral transduction of the 6 target genes, *Wt1*, *Gata4*, *Sf1*, *Sry*, *Sox9*, and *Dmrt1*, could do harm to their cell state.

To determine the influence of the protein factors on ESCs, results showed that the ESCs cultured in a medium containing 6 protein factors (mES+Protein) grew a bit faster than those without protein factors (mES+MEF and mES+Trans) (Figure 1B). Moreover, the cells grew faster in group mES+Protein+TM4 than in group mES+TM4 in the first 4-day. Supposedly, the 6 protein factors facilitated the growth of ESCs in some way. However, the growth rate immediately decreased after day 4 in group mES+Protein+TM4. It is supposed that the nutriment in the medium exhausted or the adverse metabolites had accumulated.

According to the results of growth rate, PC number, and pluripotency markers, the TM4-conditioned medium could have represented some positive influence in regulating aggregation, proliferation, and pluripotency of ESCs. However, the function of the TM4-conditioned medium still needs more evidences to determine in future research.

### Developmental Process Analysis of Non-GM-Induced eSLCs

The non-GM-induced eSLCs were derived from ESCs under the influence of the added protein factors. Without transduction of the signal sequence, these added protein factors in the culture medium can only be absorbed by endocytosis and reach where they play roles with a low chance. In comparison, lentiviral transduction brings target genes into the nucleus and produces target factors working like endogenous protein. Results indicated that the transcriptional expression level of the target genes had been greatly improved in ESCs (group mES+Trans) (Figure 1I). Since *Wt1*, *Gata4*, *Sry*, and *Sox9* were intranuclear factors in precursors of eSCs, adding recombinant protein factors had a much lower efficiency to alter the fate of ESC differentiation compared to lentiviral transduction. However, some evidences showed that these target factors worked in a rather low expression level (Buganim et al., 2012). Thus, the concentration of the recombinant protein factors in the conditioned medium was supposed to be crucial to the efficiency of inducing eSLCs.

**FIGURE 2:**
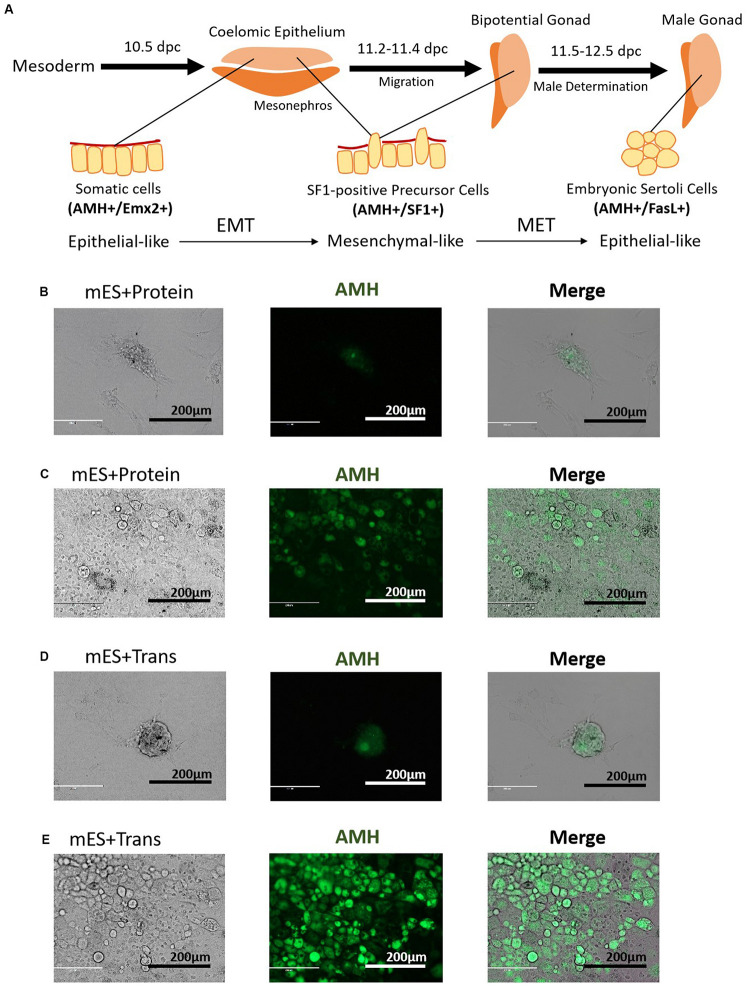
Morphology of PCs and potential progenitor cells of eSCs. **(A)** Stepwise development of progenitor cells to eSCs. PCs in panel **(B)** group mES+Protein at day 20, **(C)** epithelial-like cells in group mES+Protein, **(D)** PCs in group mES+Trans at day 20, and **(E)** epithelial-like cells in group mES+Trans express AMH (green fluorescence). Results show light microscope, IF, and merged images. Among panels **(B–E)**, scale bar = 200 μm.

**FIGURE 3:**
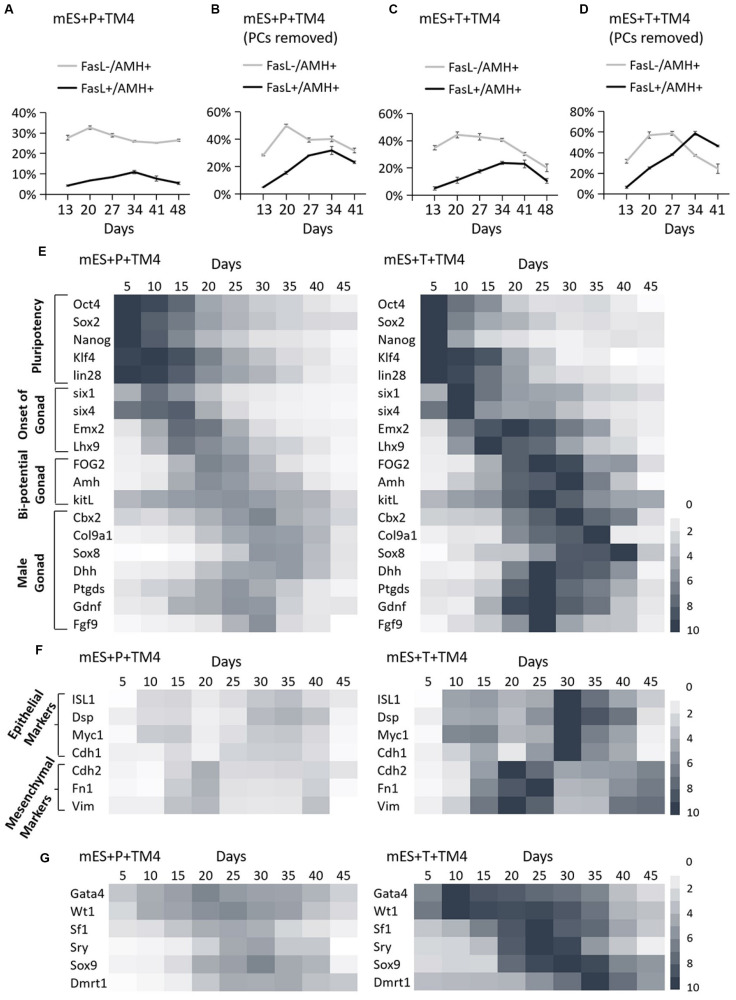
Identification of development process in group mES+P+TM4 and mES+T+TM4 by FCM and qPCR. The cell portions of FasL^–^/AMH^+^ and FasL^+^/AMH^+^ cells were detected via FCM. **(A)** The whole cell population was identified in group mES+P+TM4. **(B)** The cell population was detected in group mES+P+TM4 after PCs were removed. **(C)** The whole cell population was identified in group mES+T+TM4. **(D)** The cell population was detected in group mES+T+TM4 after PCs were removed. Tests were performed every 7-day and expressed as mean ± SD (*n* = 3 independent experiments). Heat map indicates the gene expression levels of major transcription factors for **(E)** pluripotency and onset of gonad, bi-potential gonad, and male gonad in group mES+P+TM4 and in group mES+T+TM4. **(F)** Epithelial and mesenchymal markers in group mES+P+TM4 and in group mES+T+TM4. **(G)** The inducing factors in group mES+P+TM4 and in group mES+T+TM4. qPCR was performed every 5-day and expressed relative to the highest expression value between groups mES+P+TM4 and mES+T+TM4. qPCR results took the mean value and are shown in a heat map (*n* = 3 independent experiments).

The developmental process of lentiviral transgenic-induced eSLCs could be a positive reference to non-GM-induced eSLCs and reveal the barriers. In comparison of the eSLC-generating efficiency, a specific marker, AMH, was applied since it is highly expressed during the development of eSLCs and their progenitor cells. At day 20, the PCs of group mES+Trans and mES+Protein showed a low expression of AMH (green) (Figures 2B,D). Also, the colonies of epithelial-like cells in group mES+Trans were expressed much higher than those in group mES+Protein (Figures 2C,E). Based on the results, lentiviral transduction of the 6 factors performed better in inducing AMH high expression cells than adding protein factors. Then, groups mES+P+TM4 (ESCs cultured in the TM4-conditioned medium containing 6 protein factors) and mES+T+TM4 (ESCs transduced by 6 target genes cultured in the TM4-conditioned medium) were compared according to two specific markers, AMH and FasL, through FCM. FasL^–^/AMH^+^ cells included progenitor and precursor of eSCs and potentially other mesodermal cells. Also, FasL^+^/AMH^+^ cells mainly indicated induced eSLCs in this case. Results showed there were more FasL^–^/AMH^+^ cells in mES+T+TM4 than in mES+P+TM4 in 13–34-day (Figures 3A,C). It implied that more progenitor and precursor cells could have been generated via lentiviral transduction of the 6 factors. Accordingly, at day 34, less induced eSLCs were found in mES+P+TM4 (10%, 1 × 10^5^ cells/cm^2^) than in mES+T+TM4 (23%, 1 × 10^5^ cells/cm^2^). To specifically identify the migrated somatic cells, the cell samples were detected by FCM with PCs mechanically removed (blowing and cell scraper) (Figures 3B,D). It turned out that the portion of FasL^–^/AMH^+^ cells decreased in the unaggregated cell population in mES+P+TM4 after day 20, while the portion increased in mES+T+TM4. Without PCs, eSLCs reached 31% in mES+P+TM4 (1 × 10^5^ cells/cm^2^) at day 34 and 55% in mES+T+TM4 (1 × 10^5^ cells/cm^2^). According to these results, the inducing efficiency of lentiviral transduction was much higher than that of protein factors.

To reveal the transcriptional differences between the two inducing methods, the cells of groups mES+P+TM4 and mES+T+TM4 were determined in some developmental stage-specific markers by qPCR (Figure 3E). The results were expressed relative to the highest gene expression between groups mES+P+TM4 and mES+T+TM4. The pluripotency markers, *Oct4*, *Sox2*, *Nanog*, *Klf4*, and *lin28*, were expressed in a higher level in group mES+P+TM4 before day 20 and then gradually decreased. On the contrary, these genes in group mES+T+TM4 declined rapidly after day 15. It indicated that there were more pluripotent stem cells in group mES+P+TM4 than in group mES+T+TM4 after 15-day. The marker genes indicating the onset of bi-potential gonad were expressed high in 10–20-day (Fujimoto et al., 2013). The bi-potential gonad marker genes stayed at a high expression level in 15–30-day (Bouma et al., 2007); male gonad marker genes were transcriptionally expressed mainly in 20–35-day (Colvin et al., 2001; Chaboissier et al., 2004; Franke et al., 2004; Kim et al., 2006; Yang and Han, 2010; Katoh-Fukui et al., 2012). These gonad-related specific marker genes had higher expression levels and longer duration in group mES+T+TM4 than in mES+P+TM4. These transcriptional expression results corresponded to the results of FCM, indicating there were more gonad- or coelomic epithelium-derived cells generated in mES+T+TM4 during the whole developmental process (Figures 3A,C).

Observing the morphological changes in the cell development could be helpful to deliver more information (Barrionuevo et al., 2011; Nel-Themaat et al., 2011; Chojnacka et al., 2016). The progenitor cells of eSLCs, mainly derived from somatic cells of coelomic epithelium, were epithelial-like cells (Karl and Capel, 1998; Piprek et al., 2016). The eSC precursor cells, also known as SF1^+^ gonadal precursor cells, were mesenchymal-like cells. eSCs were epithelial-like cells (Figure 2A). When cultured, immature and mature Sertoli cells show a mesenchymal-like appearance (Carre and Greenfield, 2016). Thus, during the development leading to eSCs, the cells undergo an EMT to generate SF1^+^ precursor cells, and then a MET to generate eSCs. After that, there could be another EMT for the eSCs to develop into immature and mature Sertoli cells. Based on the developmental roadmap, some epithelial and mesenchymal markers were applied to determine the development from ESCs to induced eSCs. Via qPCR, the high expression of epithelial marker genes occurred in 10–15-day and 30–35-day in group mES+P+TM4 as well as in group mES+T+TM4 (Figure 3F). The mesenchymal marker genes expressed high in group mES+P+TM4 in 15–20-day and at 40-day, and in group mES+T+TM4 in 15–25-day and 40–45-day. Therefore, in combined analysis with the FCM results, in group mES+P+TM4, the first EMT was speculated happening in 10–20-day and the SF1^+^ precursor cells were enriched around day 20 (Figures 3A,F). Then, MET happened in 20–30-day along with a generation of induced eSCs. Supposedly, another EMT happened in 30–40-day and the eSCs developed into immature Sertoli cells after 40-day. In comparison of the period of EMT and MET between mES+P+TM4 and mES+T+TM4, it is proven that the development process from ESCs to eSCs was similar between the inducing methods of lentiviral transduction and protein factors (Figures 3A,C,F).

In group mES+T+TM4, the transcriptional level of the 6 factors had been overexpressed. However, the changes in their expression level still could indicate some internal changes in the development. According to the qPCR results of group mES+P+TM4, *Gata4* and *Wt1* expressed high in 5–40-day (Figure 3G). *Sf1* increased in 15–30-day. Enhanced expression of *Sry* and *Sox9* began at day 15 and ended at day 40. *Dmrt1* mainly expressed in 20–45-day. In comparison, all of the 6 factors expressed much higher in mES+T+TM4 than in mES+P+TM4 during 5–45-day. *Gata4* and *Wt1* were upregulated earlier and higher in mES+T+TM4 since day 10. The enhanced expression of *Sox9* and *Dmrt1* lasted longer in mES+T+TM4. In groups mES+P+TM4 and mES+T+TM4, the high expression of *Wt1* and *Gata4* indicated that some coelomic epithelial somatic cells or gonad-related cells potentially were generated in 5–40-day. SF1^+^ gonad precursor cells could be generated in 15–30-day. Also, induced eSLCs were generated mainly in 15–40-day.

Via FCM (using FasL and AMH antibodies) and qPCR (developmental stage-specific marker genes, epithelial markers, and mesenchymal markers and the transcriptional level of the 6 inducing factors), the development from ESCs into induced eSLCs was clearly observed in mES+P+TM4 and mES+T+TM4. These results delivered evidences to evaluate the two inducing methods and provided information to reveal the barriers of efficient inducing eSLCs.

### Spermatogenic Function Verification

As mentioned above, non-GM eSLCs induced by protein factors could be well suited for the coculture technique. To confirm their value in use, these non-GM eSLCs were functionally verified in supporting the development of SSCs (Geens et al., 2011; Miryounesi et al., 2013; Griswold, 2018).

**FIGURE 4:**
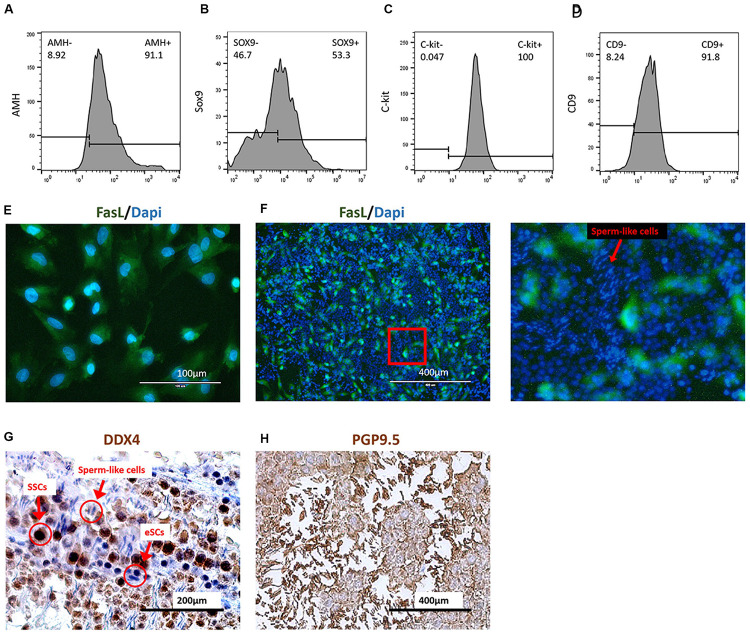
Coculture of induced eSCs with SSCs. FasL^+^ cells were sorted in group mES+P+TM4 by FCM sorter at day 30. These cells showed **(A)** 91.1% of AMH^+^ and **(B)** 53.3% of SOX9^+^ by FCM. Most of these cells were speculated as eSCs (*n* = 5. mean ± SD). SSCs were isolated by Percoll inconsecutive density gradient centrifugation. These cells showed **(C)** 100% of C-kit^+^ and **(D)** 91.8% of CD9^+^ by FCM (*n* = 5. mean ± SD). **(E)** The FasL^+^ cells sorted from mES+P+TM4 were planted in culture flasks. After 1 day, the cells were stained with DAPI (blue) and FasL antibodies (green) under IF. **(F)** Under IF, cell nuclei of the coculture of SSCs and eSCs were stained by DAPI (blue). eSCs were FasL^+^ shown in green. An enlarged image was on the right. The cells having round blue nuclei with green membrane were eSCs, and those without green membrane were supposed to be SSCs. The cells having narrow blue nuclei were speculated to be the sperm cells. **(G)** SSCs and eSCs were cocultured in SSC differentiation medium for 1 week. Via ICC, SSCs shown in dark brown as DDX4^+^ cells. eSCs and sperm-like cells were DDX4^–^ shown in blue. **(H)** SSCs and eSCs were cocultured in SSC differentiation medium for 1 week. Via ICC, SSCs and sperm-like cells are shown in dark brown as PGP9.5^+^ cells. eSCs were PGP9.5^–^ shown in blue.

The FasL^+^ cells were collected by the FCM sorter from group mES+P+TM4 after a 30-day induction. The survey sampling of these FasL^+^ cells were treated with Triton X-100 for detecting AMH (cytoplasmic) and SOX9 (intranuclear). The FCM results indicated that these FasL^+^ cells represented 90% (±3%) of AMH^+^ cells and 50% (±10%) of SOX9^+^ cells (Figures 4A,B). Under IF performed with DAPI staining and FasL antibody, these cells showed to be epithelial-like (Figure 4E). SSCs were isolated from mice according to methods described earlier. These cells were 100% (±5%) C-kit^+^ and 91.8% (±6%) CD9^+^ (Figures 4C,D).

Generally, SSCs are hard to maintain and develop into sperm without a Sertoli cell feeder (Geens et al., 2011; Baazm et al., 2017). For the coculture test, the sorted FasL^+^ cells, regarded as non-GM-induced eSLCs, were seeded and treated with mitomycin C as feeder layer at the first day. Then, SSCs were seeded at the 2nd day. After a 1-week culture, the cell nuclei were stained by DAPI and induced eSLCs showed a green label by a FasL antibody through IF (Figure 4F). Some long and narrow nuclei were observed with a brighter blue fluorescence. They were likely to be postmeiotic male gametes. ICC was performed with DDX4 antibody as a positive marker of SSCs when eSCs were DDX4^–^. According to the result, undifferentiated SSCs displayed a dark-brown color and eSLCs showed a blue color and round shape, and there were some linear cells presenting a blue color (Figure 4G). PGP9.5 were expressed in SSCs; sperm and Leydig cells in mouse testicles. Results indicated that most of these linear cells were PGP9.5^+^ (Figure 4H). Thus, these linear cells were potentially sperm-like cells. However, these sperm-like cells were not determined through valid specific markers in this work.

Based on the results, some sperm-like cells were generated in the coculture of SSCs with the non-GM-induced eSLCs derived from mES+P+TM4. The eSLCs induced by 6 protein factors still need further functional verification.

## Discussion

### A Novel Efficient Approach to Deliver Non-GM eSLCs

Sertoli cells at the embryonic stage play an important role in male determination and male gonadal development, facilitating the development of primordial germ cells (PGC) and performing better in supporting the growth of SSCs than mature Sertoli cells or the TM4 cell line (Miryounesi et al., 2013; Baazm et al., 2017). Thus, fundamental and application research on eSCs has great importance. However, it was hard to isolate adequate eSCs from donor mice for experimental performance. Moreover, the inducing efficiency and purity were not ideal in inducing Sertoli-like cells through RA and Activin A from ESCs (Bucay et al., 2009; Oeda et al., 2013; Seol et al., 2018). Therefore, we established methods to efficiently deliver non-GM eSLCs *via* TM4-conditioned medium containing six inducing protein factors, *Sry*, *Sox9*, *Sf1*, *Wt1*, *Gata4*, and *Dmrt1*. In the 35-day culture, the induced eSLCs reached 10.5% in the whole cell population (1 × 10^5^ cells/cm^2^), with 31% of PCs removed. Although the population of eSLCs induced by protein factors was less than that by lentiviral transduction (23% in whole cell population and 55% with PCs removed), these non-GM eSLCs induced by protein factors were still in a considerable number and more reliable for further experimental use.

Experimental evidence indicated that eSLCs induced by protein factors were capable of supporting the survival of SSCs. Therefore, we proposed that these non-GM eSLCs were applicable for coculture with other cells such as mesenchymal cells and neural stem cells to provide nourishing function; co-transplantation for clinical purposes to provide immune privilege (Luca et al., 2018); coculture and co-transplantation with SSCs to improve survival and development (Griswold, 2018); and developmental research to reveal the mechanism of male determination and gonadogenesis.

However, based on experimental results, there were still some barriers, such as the operation sequences of protein synthesis, purification, and renaturation, which were complicated, laborious, and error-prone; the efficiency of generating eSLCs was still low; and the induced eSLCs tend to lose markers, have a deteriorating state, or even have apoptosis over time.

The FasL^–^/AMH^+^ cells, regarded as the source of progenitor and precursor cells of eSLCs, decreased in mES+P+TM4 between 20 and 27-day, while it was still high in mES+T+TM4. As a conclusion, the lack of progenitor and precursor cells could be one of the main barriers of efficient inducing eSLCs by protein factors (Figures 3A,C). Without the signaling pathway, the protein factors passed through the cell membrane mainly by endocytosis. We supposed that the low efficiency of introduction of protein factors could be one of the main barriers for the efficient generation of induced eSLCs. To solve the issues, in the next step, we plan to apply protein transfection reagents like ProteoJuice (Novagen, German), BioTrek (Stratagene, United States), Proteinfectin Transfection Reagent (Applied Biological Materials, Canada), or ProteoCaryy (Funakoshi, Japan) to improve the efficiency. Also, future research could select other alternative factors from gene expression profiles related to gonadal development, male determination, and growth of eSLCs. Hopefully, some novel methods were promising to better deliver non-GM eSLCs.

The induced eSLCs gradually lost the expression of some specific markers including AMH, FasL, and some specific marker genes after a 41-day culture (Figures 3A–E). Thus, it was speculated that the culture condition did not meet the needs of survival or maintenance of these induced eSLCs. For a long-term culture of eSLCs, we proposed to produce a better eSLC growth medium by investigation of some growth factors and nutriments such as testosterone, EGF, β-FGF, L-ascorbic acid, RA, insulin, transferrin, selenium, ethanolamine solution (ITS-X), and progesterone (Chui et al., 2011; Mendis et al., 2011; Kulibin and Malolina, 2016; Bernardino et al., 2018). Some of these supplements were detected in gonadal development or potentially helpful in culturing Sertoli cells.

In addition, the application of the TM4-conditioned medium to culture ESCs drew some questions. TM4 cells could be in poor condition in serum-free DMEM medium for 2-day and potentially produce some harmful metabolites. Thus, the feasibility and reliability of using the TM4-conditioned medium to culture or induce ESCs need further evaluation. However, in this study, no obvious negative effect was observed through cellular morphology or qPCR result.

In conclusion, this approach we derived was a novel and efficient way to deliver non-GM eSLCs for future research in male determination, male gonad development, coculture technique, and transplantation. Moreover, it built the foundation of establishing better approaches for producing eSLCs.

### A More Reliable Cell Model for Derivation Research of eSCs

In our former research, we established a developmental roadmap from ESCs to eSLCs via lentiviral transduction of *Sry*, *Sox9*, *Sf1*, *Wt1*, *Gata4*, and *Dmrt1*. However, there still were some main barriers in modeling a developmental roadmap, *in vitro* mimicking the progress *in vivo*.

In the derivation of eSLCs via lentiviral transduction, the transcriptional expression of the target factors was greatly improved, as well as some of their downstream pathways. It brought many difficulties to precisely regulate and measure the temporal expression of the target and relevant factors. As one of the results, in the influence of genetic modification, the progenitor and precursor cells were continuously generated, differing from the development *in vivo*. For instance, *in vivo*, the precursor cells appear after 10.3 *dpc* and then develop into eSCs in 11.5–12.5 *dpc*. By contrast, via lentiviral transduction, potential precursor cells (FasL^–^/AMH^+^ cells) and eSCs (FasL^+^/AMH^+^ cells) increased in number, respectively detected in 13–27-day and 13–34-day (Figures 3C,D). Thus, the generation period of eSLCs was massively extended. Therefore, the roadmap of development from ESCs to eSCs established via lentiviral transduction has some deficiencies for male determination and male gonad development.

Here, we proposed this inducing approach to deliver non-GM eSLCs as a better development model to reveal male gonadogenesis mechanisms. qPCR results indicated that the ESCs of group mES+T+TM4 expressed high in the 6 transduced factors in the first 5-day and during the development (Figures 1I, 3G). Thus, the transcription expression of inducing factors in mES+T+TM4 was forcibly upregulated and did not accord with the development process. On the contrary, it is natural when the gene expression was not artificially overexpressed and spontaneously changed in mES+P+TM4 according to the development stages (Figures 1I, 3G). Therefore, we hold the opinion that the model established on protein factors presented more reference value. Furthermore, lentiviral transduction had the recombinant sequences inserted into an uncertain DNA site causing an unknown effect. It is more stable in the non-GM development model than in the lentivirus-established development model.

In the following, some work could be done:

(1) The efficiency was still low in this non-GM eSC-inducing method. The concentration of protein factors and transduction method should be improved. Electrotransfection by Neon Transfection System (Thermo) could be tested.
(2) The ESCs could be induced into IM-derived cells before the induction of eSLCs to improve the efficiency.
(3) The induced eSLCs need much more evidence to identify their characteristic and function.
(4) The mechanism of non-GM induction of eSLCs should be determined.

## Conclusion

Conclusively, it is a novel non-GM method to provide induced eSLCs with recombinant protein of differentiation-related factors *Wt1*, *Gata4*, *Sry*, *Sox9*, *Sf1*, and *Dmrt1*, rather than traditional factors such as FSH. These induced eSLCs are considered as a superior developmental model to mimic the generation of eSCs *in vivo*. Moreover, the non-GM-induced eSLCs hold promise to reveal the mechanism of deriving eSCs, male determination, and male gonadal development with more credible and reliable experimental results.

## Data Availability Statement

All datasets presented in this study are included in the article/Supplementary Material.

## Ethics Statement

The animal study was reviewed and approved by Permit for use of laboratory animals (SYXK) (China).

## Author Contributions

MG and CX designed the study. CX performed most experiments with help from YL, LX, QW, and AM. CX analyzed the experimental data. CX, AM, and MG wrote the manuscript. MG, HH, and YZ participated in the data discussion. All authors contributed to the article and approved the submitted version.

## Conflict of Interest

The authors declare that the research was conducted in the absence of any commercial or financial relationships that could be construed as a potential conflict of interest.

## Abbreviations

ANOVA: analysis of variance
A-P: anterior-to-posterior
BSA: bovine serum albumin
EMT: epithelial-mesenchymal transformation
eSCs: embryonic Sertoli cells
ESCs: embryonic stem cells
FBS: fetal calf serum
FCM: flow cytometry
FSH: follicle-stimulating hormone
ICC: immunocytochemistry
IF: immunofluorescence
IGF1: insulin-like growth factor 1
LIF: leukemia inhibitory factor
MEFs: mouse emrbyo fibroblasts
MET: mesenchymal-epithelial transformation
MIS: anti-Mullerian hormone
NEAA: nonessential amino acids
Non-GM: non-genetically modified
PCs: pebble-like colonies
qPCR: quantitative real time polymerase chain reaction
SD: standard deviation
Sf1: steroidogenic factor 1
SPSS: Statistical Package for Science and Society
WB: western Blot.
