# Supplementary material for: Inducing Non-genetically Modified Induced Embryonic Sertoli Cells Derived From Embryonic Stem Cells With Recombinant Protein Factors
Source: Front Cell Dev Biol. 2021 Jan 25;8:533543. doi: 10.3389/fcell.2020.533543 (PMC7875124; doi:10.3389/fcell.2020.533543)
Supplement: Supplementary file 1 [file Table_1.DOCX]

**SUPPLEMENTAL TABLES**

**Table S1 Antibodies for WB**

| Antibodies | Descripition | Producer |
| --- | --- | --- |
| **GATA4 antibody**[orb216114] | Rabbit polyclonal | Biorbyt |
| **WT1 antibody**[orb29938] | Rabbit polyclonal | Biorbyt |
| **NR5A1 antibody**[orb35403] | Rabbit polyclonal | Biorbyt |
| **SOX9 antibody [orb4387]** | Rabbit polyclonal | Biorbyt |
| **DMRT1 antibody [orb47259]** | Rabbit polyclonal | Biorbyt |
| **Goat Anti-Rabbit IgG antibody**[orb27745] | Secondary antibody | Biorbyt |
| **SRY antibody (D-11): sc-398567** | Mouse monoclonal | Santa Cruz |
| **m-IgGκ BP-HRP: SC-516102** | Secondary antibody | Santa Cruz |

WB (Western Blot)

**Table S2 Primers for complete gene amplification of the target factors**

| Primer name | Forward | Reverse | Length（bp） |
| --- | --- | --- | --- |
| *Sry* | CGGGATCCCGTACCTCCCGGTACAGTTCGC | GGAATTCCTCATGAGACTGCCAACCACA | 1188 |
| *Sox9* | CGGGATCCCGACTCGACCTTCAGCCTCTCG | GGAATTCCTGTTTTCACTTTAATGCAAT | 4146 |
| *Sf1* | CGGGATCCCGTCAGGCGCGTCATTGTTCCT | GGAATTCCGGCTGAGCCATGGTTAAGTC | 2994 |
| *Wt1* | CGGGATCCCGACACACTTACCTCGCCGGCT | GGAATTCCTTCCTTTTGAATAGACTTTA | 3092 |
| *Gata4* | CGGGATCCCGCGCGGCCCTCGTCCCCTGTT | GGAATTCCTCGGAGGAAACAGGAATACA | 3408 |
| *Dmrt1* | CGGGATCCCGTCTTTTTTCTTTTTTCTTTT | GGAATTCCGCAGTCATCAATAACGTTTT | 2251 |

**Table S3 Primers for qRT-PCR (quantitative realtime polymerase chain reaction)**

| Primer name | Forward primer | Reverse primer | Length（bp） |
| --- | --- | --- | --- |
| *Oct4* | GGCTTCAGACTTCGCCTTCT | TGGAAGCTTAGCCAGGTTCG | 99 |
| *Sox2* | CAAAAACCGTGATGCCGACT | CGCCCTCAGGTTTTCTCTGT | 92 |
| *Nanog* | AGCCGTTGGCCTTCAGATAG | AAGTCAGAAGGAAGTGAGCCG | 82 |
| *Klf4* | TACCCCTACACTGAGTCCCG | GGAAAGGAGGGTAGTTGGGC | 110 |
| *lin28* | CTTTGCCTCCGGACTTCTCT | AAACTGCTGGTTGGACACCG | 101 |
| *six1* | CACGCCAGGAGCTCAAACTA | ACCCAAGTCCACCAAACTGG | 132 |
| *six4* | CCCCACCGGGCAGATTG | GTCCTTCCGAGGCGCTTT | 71 |
| *Lhx9* | GGACCGCTGAATCTAGCACT | CTGCAGCGAGAGATCGAGTT | 141 |
| *Emx2* | GGTTTCAGAACCGGAGAACG | CTATTTCCTCCGGACTCGCC | 138 |
| *Gata4* | AGCAGGACTCTTGGAACAGC | GCCCCAGCCTTTTACTTTGC | 138 |
| *Wt1* | ATCCCAGGCAGGAAAGTGTG | GTGCTGTCTTGGAAGTCGGA | 111 |
| *Sf1* | GCCCGAGTGGCCGTC | TGTCACCACACACTGGACAC | 148 |
| *Sry* | TGGTGAGAGGCACAAGTTGG | AGGCTTTTCCACCTGCATCC | 87 |
| *Sox9* | GTGCAAGCTGGCAAAGTTGA | TGCTCAGTTCACCGATGTCC | 106 |
| *Dmrt1* | GGATCCCCCGTGAAGAACAG | GAGGCCCGTAGTATGAGTGC | 142 |
| *FOG2* | CCTAACTGAAGAAGCCGCCA | AGGCGCACATATAGCAGTCC | 105 |
| *Sox8* | GATACATCCAACCCGGGGAC | GCTGGGAAGAACGGTGGTAA | 76 |
| *Dhh* | TGGCTTGGGTGTATCTGTGG | AACGAGACCGGGAAAGAACG | 111 |
| *ISL1* | ATCGAGTGTTTCCGCTGTGT | AAGGGACTGAGAGGGTCTCC | 146 |
| *Nanog* | AGCCGTTGGCCTTCAGATAG | AAGTCAGAAGGAAGTGAGCCG | 82 |
| *Amh* | CTACTCAAGGACAGCTCAGGC | GGAGGCGACTGTCTCAAAGG | 78 |
| *kitL* | CAGCGCTGCCTTTCCTTATG | TCCCGGAGCGATTTTCTTGG | 89 |
| *Ptgds* | GGCTCCTTCTGCCCAGTTTTC | CCAGGAGGACCAAACCCATC | 120 |
| *Gdnf* | TGTTCCGCGCTTCTTCTTCT | TCCTTCCTCCTCCGAGTGTC | 120 |
| *Fgf9* | ACGGTCGGATGGGATGAAGA | TGGCACAGGTTCAAGGTCAA | 147 |
| *Cbx2* | GGAGTACCTGGTCAAGTGGC | GCCTCGGGTCCAAAATGTTC | 80 |
| *Col9a1* | AAGAAGGAGCAAGCTTGGGG | CCAGTGGGTTCTCTGTGGTT | 125 |

**Table S4 Lentivirus producing and packaging system**

| Size | FUW-TetO + target gene（μg） | PAX2  （μg） | PMD2.G  （μg） |
| --- | --- | --- | --- |
| 24 well plate | 0.25 | 0.187 | 0.062 |
| 12 well plate | 0.5 | 0.375 | 0.125 |
| 6 well plate | 1 | 0.75 | 0.25 |
| T25 flask | 2 | 1.5 | 0.5 |
| T75 flask | 6 | 4.5 | 1.5 |

**Table S5 Antibodies for IF and ICC**

| Antibodies | Descripition | Producer |
| --- | --- | --- |
| FasL antibody（Kay-10）: sc-19988 | mouse monoclonal IgG_2b_ (kappa light chain)  Application: IF, ICC and FCM | Santa Cruz |
| Anti-AMH antibody C -terminal: ab229212 | Rabbit polyclonal  Application: IF, ICC | Abcam |
| m-IgGk BP-HRP | mouse IgGk binding protein-HRP  Application: ICC | Santa Cruz |
| m-IgGk BP-FITC:  sc-516140 | mouse IgG kappa binding protein conjugated to fluorescein (FITC)  Application: IF, FCM | Santa Cruz |
| mouse anti-rabbit IgG-CFL 488: sc-516248 | mouse monoclonal secondary antibody conjugated to CruzFluor^TM^ 488  Application: IF, ICC, FCM | Santa Cruz |

IF (Immunofluorescence); ICC (Immunocytochemistry); FCM (Flow cytometry)

**Table S6 Antibodies for development stage identification by FCM**

| Antibodies | Descripition | Producer |
| --- | --- | --- |
| AMH antibody: orb40644 | Primary antibody: Rabbit polyclonal unconjugated | Biorbyt |
| CD178 (Fas Ligand) (MFL3) | Primary antibody: Armenian hamster monoclonal conjugated to APC | eBioscience |
| Anti-SOX9 antibody (ab185966) | Primary antibody: Rabbit monoclonal [EPR14335-78] | Abcam |
| Mouse anti-rabbit IgG-FITC: sc-2359 | Second antibody: Mouse monoclonal conjugated to FITC | Santa Cruz |
| CD117 (c-Kit) (2B8) | Primary antibody: Rat monoclonal (MFL3) conjugated to PE | eBioscience |
| CD9 (eBioKMC8 (KMC8)) | Primary: Rat Monoclonal Antibody conjugated to FITC | eBioscience |

FCM (Flow cytometry)
